# Supplementary material for: C9orf72-derived arginine-rich poly-dipeptides impede phase modifiers
Source: Nat Commun. 2021 Sep 6;12:5301. doi: 10.1038/s41467-021-25560-0 (PMC8421406; doi:10.1038/s41467-021-25560-0)
Supplement: Supplementary file 1 — Supplementary Information [file 41467_2021_25560_MOESM1_ESM.pdf]

Supplementary Information

C9orf72-derived arginine-rich poly-dipeptides impede phase modifiers

Hitoki Nanaura[1], Honoka Kawamukai[1], Ayano Fujiwara[1], Takeru Uehara, Yuichiro Aiba, Mari Nakanishi, Tomo Shiota, Masaki Hibino, Pattama Wiriyasermkul, Sotaro Kikuchi, Riko Nagata, Masaya Matsubayashi, Yoichi Shinkai, Tatsuya Niwa, Taro Mannen, Naritaka Morikawa, Naohiko Iguchi, Takao Kiriyama, Ken Morishima, Rintaro Inoue, Masaaki Sugiyama, Takashi Oda, Noriyuki Kodera, Sachiko Toma-Fukai, Mamoru Sato, Hideki Taguchi, Shushi Nagamori, Osami Shoji, Koichiro Ishimori, Hiroyoshi Matsumura, Kazuma Sugie, Tomohide Saio*, Takuya Yoshizawa*, Eiichiro Mori*

[1]These authors contributed equally: Hitoki Nanaura, Honoka Kawamukai, Ayano Fujiwara.

*Corresponding authors.

E-mail: saio@tokushima-u.ac.jp, t-yosh@fc.ritsumei.ac.jp, emori@naramed-u.ac.jp.

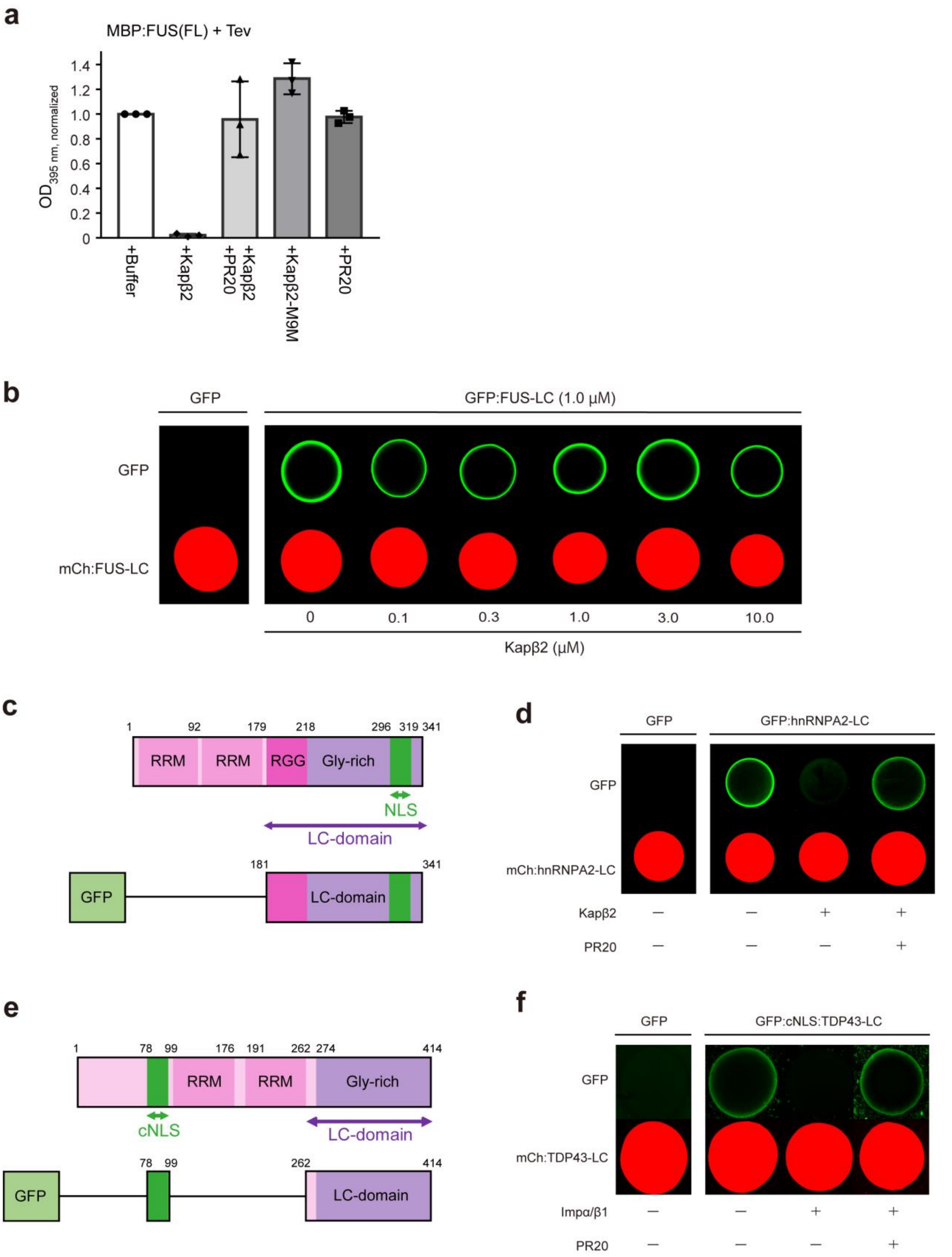

a
MBP:FUS(FL) + Tev
OD395 nm, normalized
1.4
1.2
1.0
0.8
0.6
0.4
0.2
0
+Buffer
+Kapβ2
+Kapβ2 +PR20
+Kapβ2-M9M
+PR20
b
GFP
GFP:FUS-LC (1.0 µM)
GFP
mCh:FUS-LC
0
0.1
0.3
1.0
3.0
10.0
Kapβ2 (µM)
c
1
92
179
218
296
319
341
RRM
RRM
RGG
Gly-rich
NLS
LC-domain
181
341
GFP
LC-domain
d
GFP
GFP:hnRNPA2-LC
GFP
mCh:hnRNPA2-LC
Kapβ2
PR20
e
1
78
99
176
191
262
274
414
RRM
RRM
Gly-rich
cNLS
LC-domain
78
99
262
414
GFP
LC-domain
f
GFP
GFP:cNLS:TDP43-LC
GFP
mCh:TDP43-LC
Impα/β1
PR20

Supplementary Fig. 1. Domain architecture and hydrogel binding assay of RNA binding proteins. **a)** Turbidity of 8 µM MBP:FUS in the presence of buffer, ± 8 µM Kapβ2, ± 8 µM Kapβ2-M9M complex, and ± 8 µM PR20. OD 395 nm is normalized to measurement of MBP:FUS + buffer + Tev. Mean of three technical replicates, ±SD. **b)** Hydrogel droplets of mCh:FUS-LC (lower images) were incubated with 1.0 µM of GFP (left panel) or 1.0 µM of GFP:FUS-LC (right panel) and visualized by confocal microscopy. GFP:FUS-LC was challenged for homotypic polymer extension in the presence of different concentration of Kapβ2 (left to right: 0.1 µM, 0.3 µM, 1.0 µM, 3.0 µM, 10.0 µM, respectively). **c)** Domain architecture of hnRNPA2 and GFP fusion hnRNPA2-LC (GFP:hnRNPA2-LC). NLS of hnRNPA2 (residue 296-319) is located in the middle of LC-domain. **d)** mCh:hnRNPA2-LC (lower images) were incubated with 1.0 µM of GFP (left panel) or 1.0 µM of GFP:hnRNPA2-LC (right panel). GFP:hnRNPA2-LC containing Kapβ2 (1.0 µM) was challenged in the absence or presence of PR20 (1.0 µM**). e)** Domain architecture of TDP43 and GFP-tagged cNLS fusion TDP43-LC (GFP:cNLS:TDP43-LC). **f)** mCh:TDP43-LC (lower images) were incubated with 1.0 µM of GFP (left panel) or 1.0 µM of GFP:cNLS:TDP43-LC (right panel) in the absence or presence of Impα/β1 (1.0 µM/1.0 µM) and/or PR20 (1.0 µM). Source data are provided as a Source Data file. RGG: Arg-Gly-Gly motif, RRM: RNA Recognition Motif, ZnF: zinc-finger domain.

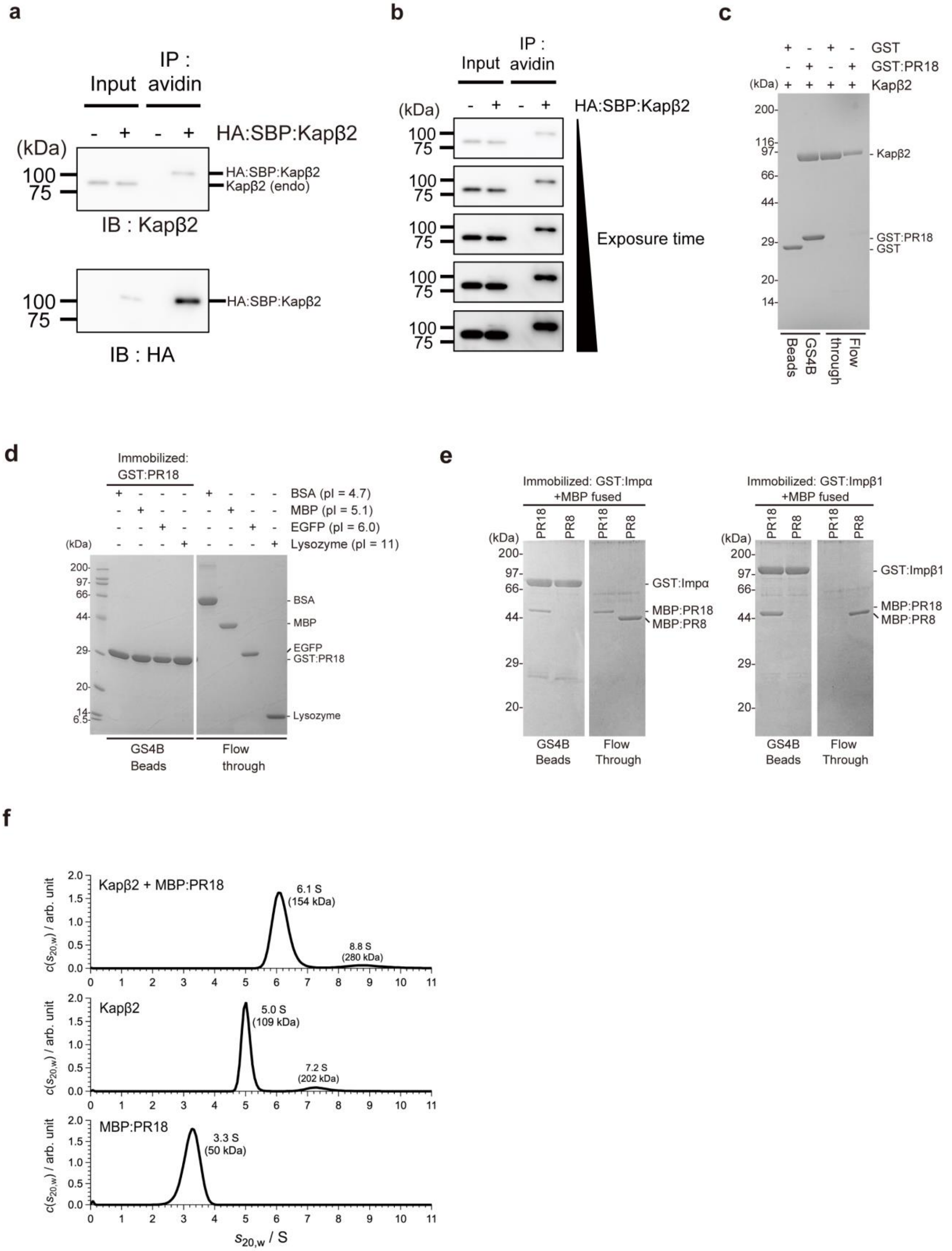
a
IP : avidin
Input
- + - + HA:SBP:Kapβ2
(kDa)
100
75
HA:SBP:Kapβ2
Kapβ2 (endo)
IB : Kapβ2
HA:SBP:Kapβ2
IB : HA
b
Input
IP : avidin
(kDa)
HA:SBP:Kapβ2
Exposure time
c
GST
GST:PR18
Kapβ2
Kapβ2
GST:PR18
GST
GS4B Beads
Flow through
d
Immobilized: GST:PR18
BSA (pI = 4.7)
MBP (pI = 5.1)
EGFP (pI = 6.0)
Lysozyme (pI = 11)
BSA
MBP
EGFP
GST:PR18
Lysozyme
GS4B Beads
Flow through
e
Immobilized: GST:Impα +MBP fused
Immobilized: GST:Impβ1 +MBP fused
PR18
PR8
GST:Impα
GST:Impβ1
MBP:PR18
MBP:PR8
GS4B Beads
Flow Through
f
Kapβ2 + MBP:PR18
6.1 S (154 kDa)
8.8 S (280 kDa)
Kapβ2
5.0 S (109 kDa)
7.2 S (202 kDa)
MBP:PR18
3.3 S (50 kDa)
c(s20,w) / arb. unit
s20,w / S

Supplementary Fig. 2. Interaction between nuclear import receptors and PR poly-dipeptides. **a-b)** Immunoprecipitation showing the recognition of endogenous and exogenous Kapβ2 by anti-Kapβ2 antibody. **c)** Pull-down binding assay showing interaction between GST:PR18 and Kapβ2. **d)** Pull-down binding assay showing no interaction between GST:PR18 and proteins, BSA, MBP, EGFP, and Lysozyme. **e)** Pull-down binding assay showing interaction between MBP:PR18 with GST:Importinα or GST:Importinβ1. **f)** Analytical ultracentrifugation showing complex formation between Kapβ2 and MBP:PR18. The experiments (**a**, **c**, **e**) were independently repeated two or three times with similar results. Source data are provided as a Source Data file.

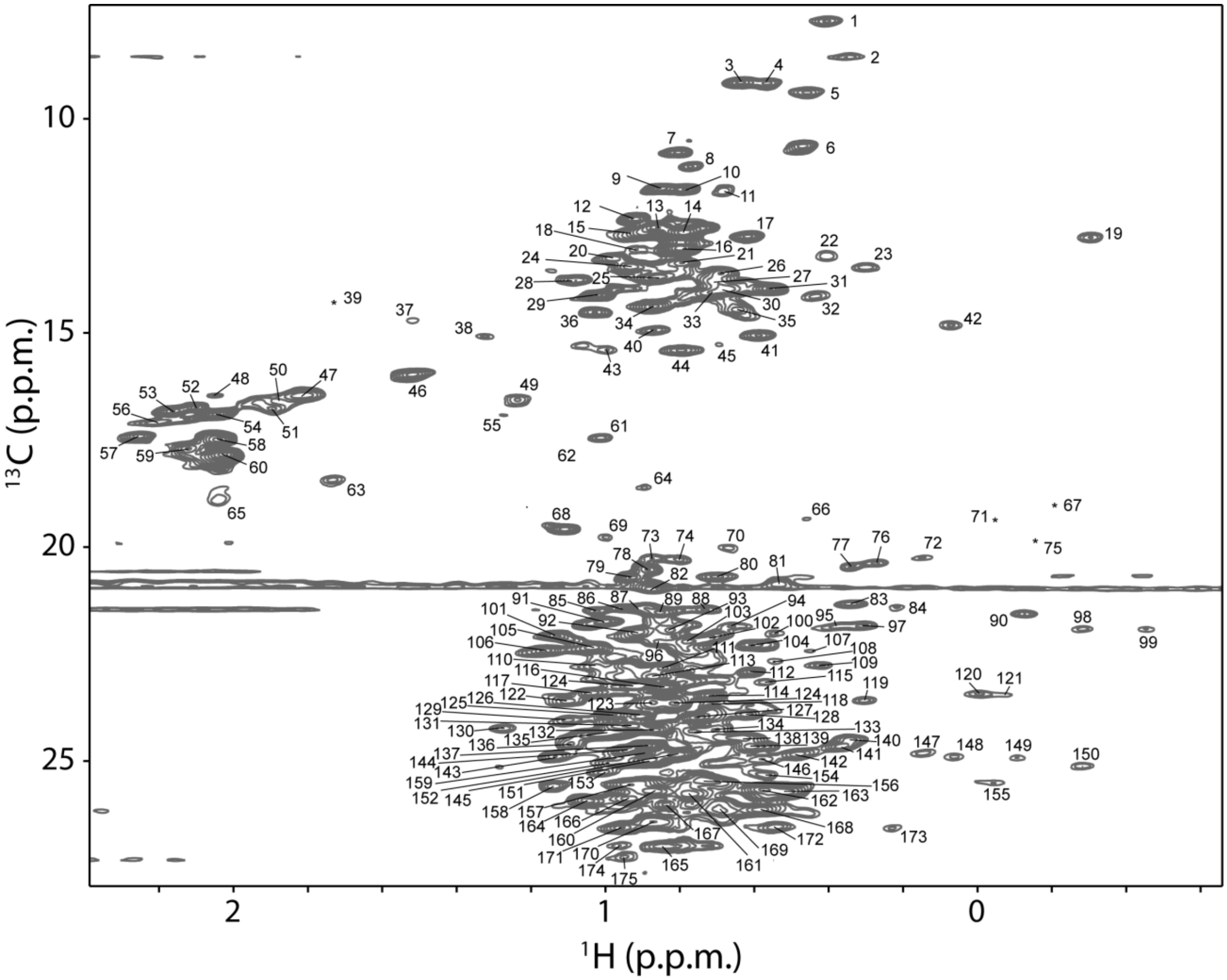

Supplementary Fig. 3. Solution NMR of Kapβ2. ^{1}H-^{13}C-correlated methyl NMR spectra of [U-^{2}H; Ile-1-$^{13}CH_3$; Leu, Val-$^{13}CH_3/^{12}CH_3$]-labeled Kapβ2. The peaks are labeled with peak numbers. Asterisks indicate the positions of the small peaks that were absent in the threshold setting of the figure.

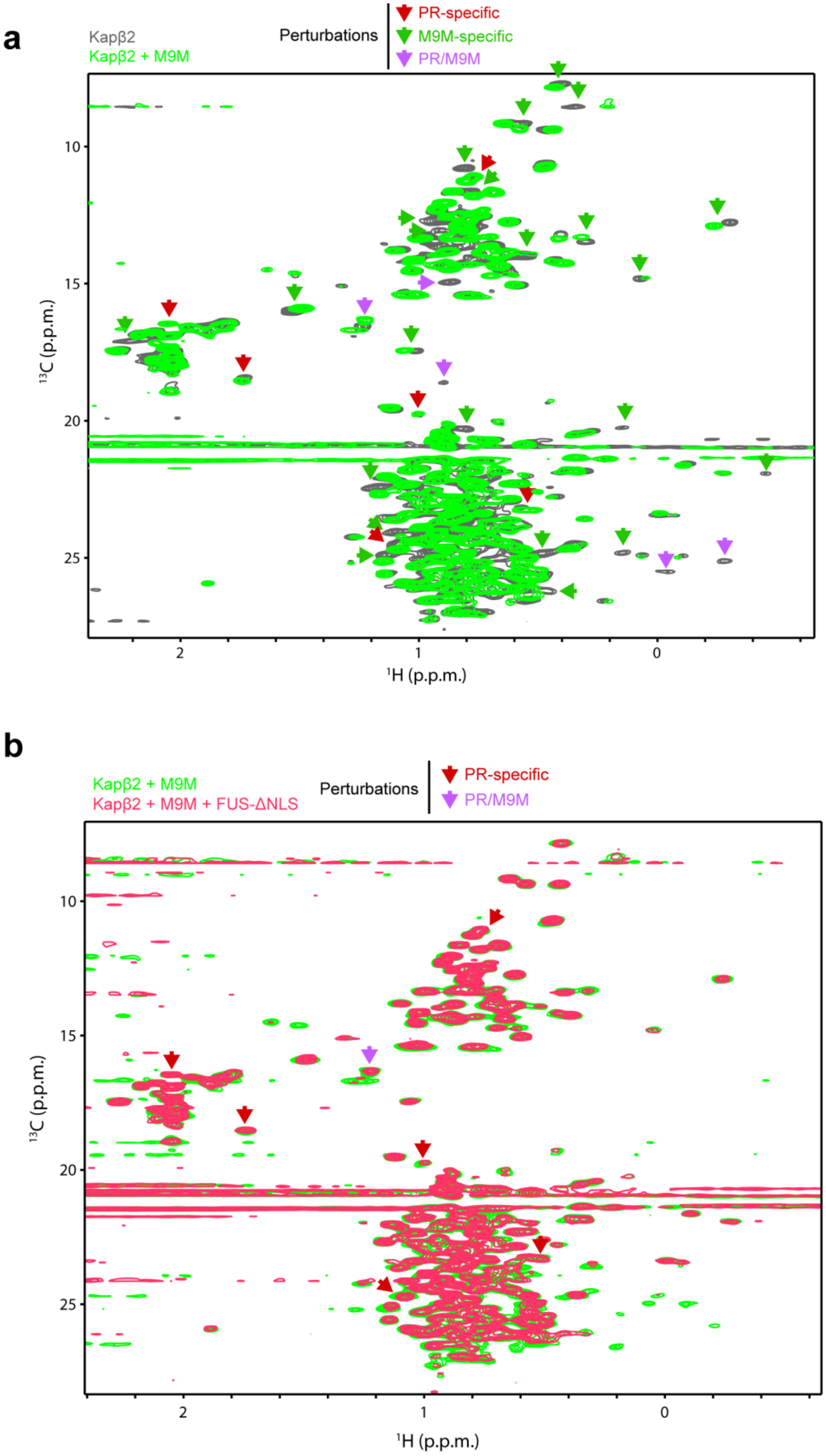
a
Kapβ2
Kapβ2 + M9M
Perturbations
PR-specific
M9M-specific
PR/M9M
10
15
20
25
2
1
0
^{13}C (p.p.m.)
^{1}H (p.p.m.)
b
Kapβ2 + M9M
Kapβ2 + M9M + FUS-ΔNLS
Perturbations
PR-specific
PR/M9M
10
15
20
25
2
1
0
^{13}C (p.p.m.)
^{1}H (p.p.m.)

Supplementary Fig. 4. Interaction between Kapβ2 and M9M/FUS-ΔNLS investigated by solution NMR. a) ^{1}H-^{13}C-correlated methyl NMR spectra of [U-^{2}H; Ile-1-$^{13}CH_3$; Leu, Val-$^{13}CH_3$/$C^{2}H_3$]-labeled Kapβ2 (grey) and that in complex with M9M (green). The significant perturbations were observed, which is consistent with the high affinity between Kapβ2 and M9M as reported in a previous study (Supplementary Table 1). **b)** ^{1}H-^{13}C-correlated methyl NMR spectra of [U-^{2}H; Ile-1-$^{13}CH_3$; Leu, Val-$^{13}CH_3$/$^{12}CH_3$]-labeled Kapβ2 in complex with M9M, in the absence (green) and presence (magenta) of FUS-ΔNLS. Significant representative perturbations are indicated by arrows. Perturbations only seen for PR20 are indicated by red arrows. Perturbations only seen for M9M are indicated by green arrows. Perturbations common to PR20 and M9M are indicated by the purple arrows. Perturbed resonances induced by the addition of FUS-ΔNLS were distinct from those induced by PR20.

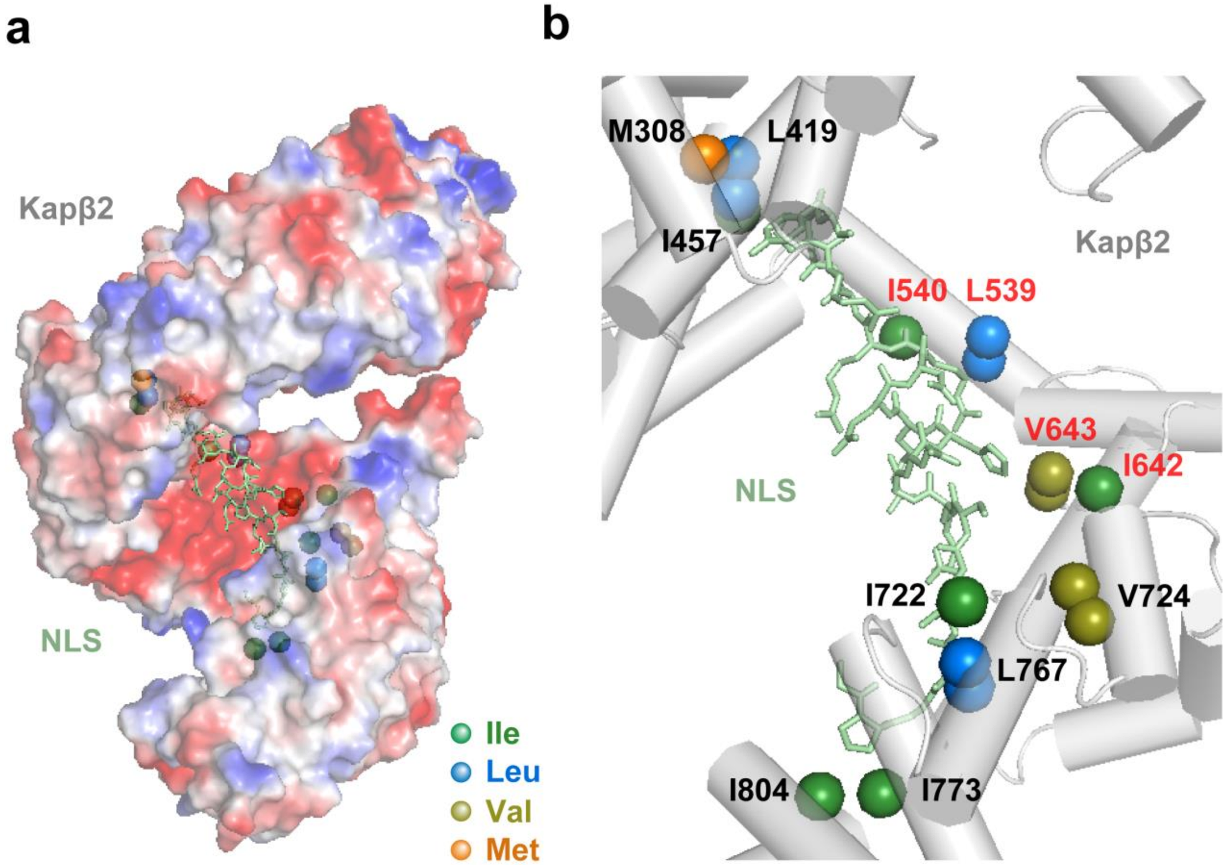

Supplementary Fig. 5. Structural model of Kapβ2 and NLS of FUS. a) Electrostatic surface potential of Kapβ2 (4FDD). Positive and negative surface potentials are drawn in blue and red, respectively. The methyl groups of isoleucine, leucine, valine, and methionine residues located close to the Kapβ2 cavity are represented as spheres colored green, blue, yellow, and orange, respectively. **b)** An expanded view of Kapβ2 cavity. Methyl groups of isoleucine, leucine, valine, and methionine are represented as spheres colored green, blue, yellow, and orange, respectively. L539, I540, V643, and I642 are located on negatively charged cavity. NLS of FUS is represented as a stick model, colored pale green. The structure was drawn using PyMol program.

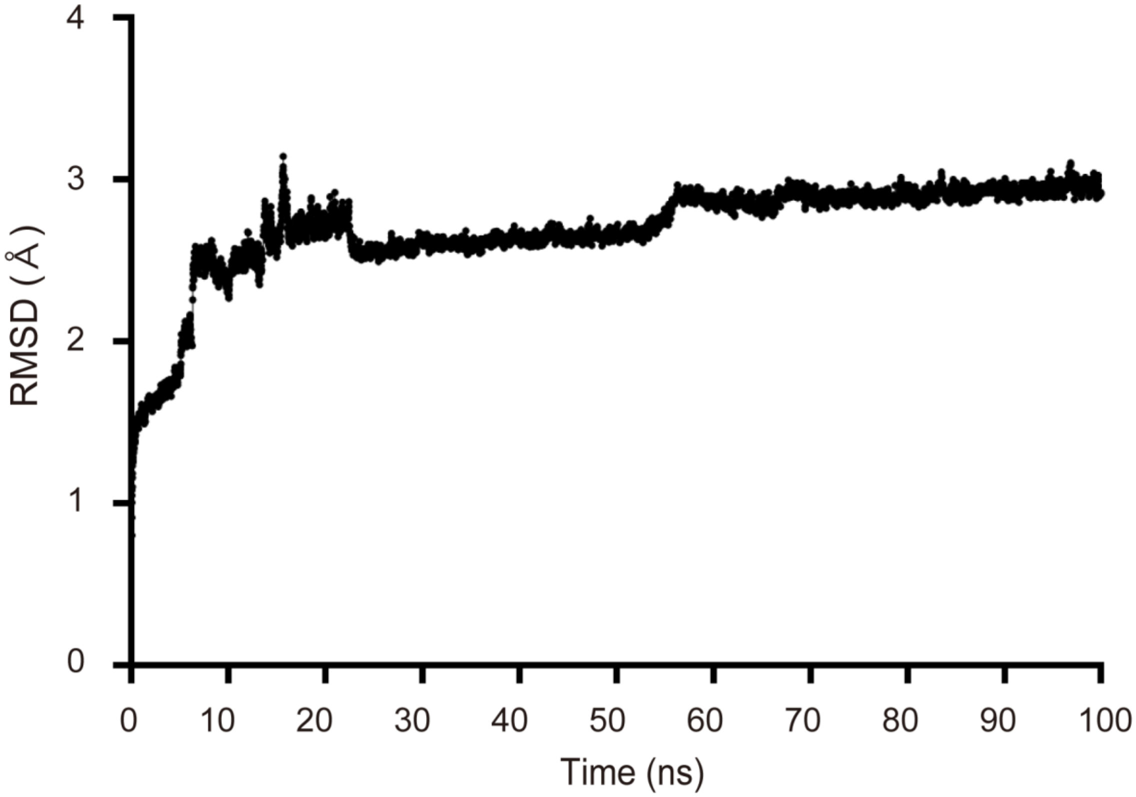

Supplementary Fig. 6. RMSD values of all atoms from the reference structure (0 ns) for MD simulation. Root-mean-square deviations (RMSDs) of all atoms were plotted as a function of time for the 100-ns MD simulation (Fig. 5a, b) of Kapβ2 and PR poly-dipeptides. Source data are provided as a Source Data file.

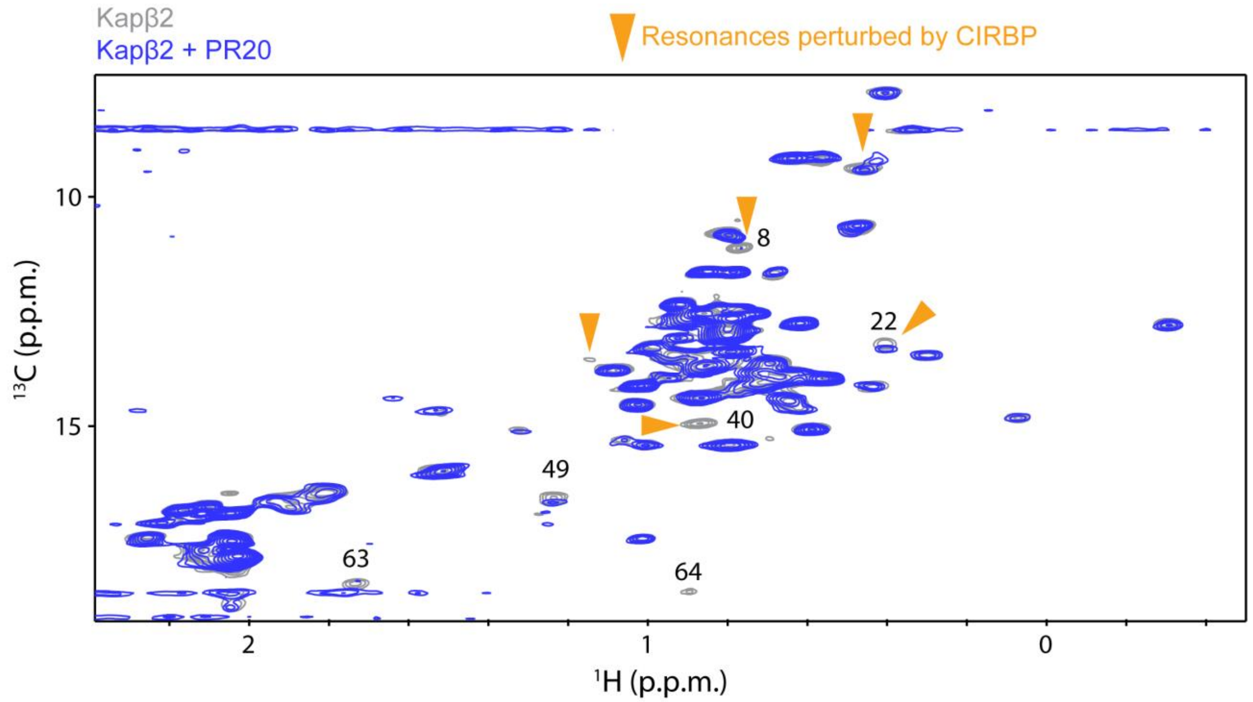

Supplementary Fig. 7. Comparison of NMR perturbations of Kapβ2 induced by PR20 and CIRBP-RGG. ^{1}H-^{13}C-correlated methyl NMR spectra of [U-^{2}H; Ile-1-$^{13}CH_3$; Leu, Val-$^{13}CH_3$/$C^{2}H_3$]-labeled Kapβ2 in the absence (grey) and presence (blue) of PR20. The resonances that showed significant perturbations by interaction with CIRBP-RGG[1] are indicated by arrowheads.

Supplementary Table 1. Kd value against Kapβ2 determined by ITC

Ligand	Tag	Kd (nM)	Reference	Buffer
PR18	MBP	81.3	This work	20 mM HEPES pH7.4, 150 mM NaCl, 10% glycerol, 2 mM bME
FUS(475-526)	MBP	31	Gonzalez et al., Sci Rep, 2021	20 mM Tris-HCl pH7.4, 150 mM NaCl, 10% Glycerol, 2 mM bME
FUS(453-500)	MBP	6000	Gonzalez et al., Sci Rep, 2021	20 mM Tris-HCl pH7.4, 150 mM NaCl, 10% Glycerol, 2 mM bME
FUS(371-500)	MBP	170	Gonzalez et al., Sci Rep, 2021	20 mM Tris-HCl pH7.4, 150 mM NaCl, 10% Glycerol, 2 mM bME
FUS(1-500)	MBP	160	Gonzalez et al., Sci Rep, 2021	20 mM Tris-HCl pH7.4, 150 mM NaCl, 10% Glycerol, 2 mM bME
FUS(415-460)	MBP	No binding	Gonzalez et al., Sci Rep, 2021	20 mM Tris-HCl pH7.4, 150 mM NaCl, 10% Glycerol, 2 mM bME
FUS(371-452)	MBP	4000	Gonzalez et al., Sci Rep, 2021	20 mM Tris-HCl pH7.4, 150 mM NaCl, 10% Glycerol, 2 mM bME
FUS(278-385)	MBP	No binding	Gonzalez et al., Sci Rep, 2021	20 mM Tris-HCl pH7.4, 150 mM NaCl, 10% Glycerol, 2 mM bME
FUS(1-370)	MBP	35000	Gonzalez et al., Sci Rep, 2021	20 mM Tris-HCl pH7.4, 150 mM NaCl, 10% Glycerol, 2 mM bME
FUS(476-526)	MBP	71.5	Yoshizawa et al., Cell, 2018	20 mM HEPES pH7.4, 150 mM NaCl, 10% glycerol, 2 mM bME
FUS(453-526)	MBP	173	Yoshizawa et al., Cell, 2018	20 mM HEPES pH7.4, 150 mM NaCl, 10% glycerol, 2 mM bME
FUS(371-526)	MBP	209	Yoshizawa et al., Cell, 2018	20 mM HEPES pH7.4, 150 mM NaCl, 10% glycerol, 2 mM bME
FUS(1-526)	MBP	158.7	Yoshizawa et al., Cell, 2018	20 mM HEPES pH7.4, 150 mM NaCl, 10% glycerol, 2 mM bME
M9NLS(A1, 257-305)	MBP	42	Lee et al., Cell, 2006	20 mM Tris pH7.5, 100 mM NaCl, 2 mM bME
hnRNP M(41-70)	MBP	10	Cansizoglu et al., NSMB, 2007	20 mM Tris pH7.5, 100 mM NaCl, 2 mM bME
M9M	MBP	0.107	Cansizoglu et al., NSMB, 2007	20 mM Tris pH7.5, 100 mM NaCl, 2 mM bME
FUS(454-526)	–	600	Dormann et al., EMBO J, 2012	20 mM sodium phosphate buffer pH6.8, 50 mM NaCl. 1 mM EDTA, 1 mM DTT
FUS(489-526)	–	2800	Dormann et al., EMBO J, 2012	20 mM sodium phosphate buffer pH6.8, 50 mM NaCl. 1 mM EDTA, 1 mM DTT
FUS(504-526)	–	20000	Dormann et al., EMBO J, 2012	20 mM sodium phosphate buffer pH6.8, 50 mM NaCl. 1 mM EDTA, 1 mM DTT
FUS(473-503)	–	11700	Dormann et al., EMBO J, 2012	20 mM sodium phosphate buffer pH6.8, 50 mM NaCl. 1 mM EDTA, 1 mM DTT

Supplementary Table 2. Primers for plasmid construction

Name	Sequence
pGEX_PR_F	CCACGCCCACGCCCACGCCCACGCCCACGCCCACGCCCACGCCCACGCCCACGC<br>CCACGCTAACTCGAGCGGCCGCATCGTGACTGACTG
pGEX_PR_R	ACGCGGACGCGGACGCGGACGCGGACGCGGACGCGGACGCGGACGCGGACGCGG<br>ACGCGGGGATCCCAGGGGCCCCTGGAACAGAACTTC
pMAL_DPR_F	GGAGAAAATCTTTATTTTCAAGGTCTGGAAGTTCTGTTCCAGGGGCCCCTGGGA
pMAL_DPR_R	GGATCCCTCGAGGAATTCGGTACCCAGTCAGTCACGATGCGGCCGCTCGAGTTA
pMAL_vec_F	GGTACCGAATTCCTCGAGGGATCCTCTAGA
pMAL_vec_R	ACCTTGAAAATAAAGATTTTCTCCCCTTCC
FUS-NLS_inf_F	CAGCAGGACCGTGGAGGGGACAGAGGTGGCTT
FUS-NLS_inf_R	GTTAGCAGCCGGATCTTAATACGGCCTCTCCCTGCG
pHis-GFP_FUS-LC_line_F	GATCCGGCTGCTAACAAAGCC
pHis-GFP_FUS-LC_line_R	TCCACGGTCCTGCTGTC
TDP43-LC_F	AACCCAAGCACAACAGCAATCG
TDP43-LC_R	ACATGCCCCAGCCAGAGCTCT
TDP43-LC_inf_F	CATGGATCCGGAATTACCCAAGCACAACAGCAATCG
TDP43-LC_inf_R	AGTTGAGCTCGTCGAATTACATGCCCCAGCCAGAG
pHis-GFP_line_F	CCCAAGCACAACAGCAATCG
pHis-GFP_line_R	TAATTCCGGATCCATGGTACCCG
TDP43-cNLS_inf_F	ATGGATCCGGAATTACCCAAGGACAACAAGCGC
TDP43-cNLS_inf_R	GCTGTTGTGCTTGGGTGCGCGCTTGACCTTCACTGC
HA-SBP_F	CTTAAGCTTCACCATGTACCCATACGATGTTCCAG
HA-SBP_R	AGTGGATCCGGGCTCCCGCTGGCCCTGAG

Supplementary References

1. Bourgeois B*, et al.* Nonclassical nuclear localization signals mediate nuclear import of CIRBP. *Proc Natl Acad Sci USA* **117**, 8503-8514 (2020).
