# Supplementary material for: C9orf72-derived arginine-rich poly-dipeptides impede phase modifiers
Source: Nat Commun. 2021 Sep 6;12:5301. doi: 10.1038/s41467-021-25560-0 (PMC8421406; doi:10.1038/s41467-021-25560-0)
Supplement: Supplementary file 3 — Reporting summary [file 41467_2021_25560_MOESM3_ESM.pdf]

nature research

Corresponding author(s): Tomohide Saio
Takuya Yoshizawa
Eiichiro Mori

Last updated by author(s): Aug 3, 2021

Reporting Summary

Nature Research wishes to improve the reproducibility of the work that we publish. This form provides structure for consistency and transparency in reporting. For further information on Nature Research policies, see our Editorial Policies and the Editorial Policy Checklist.

Statistics

For all statistical analyses, confirm that the following items are present in the figure legend, table legend, main text, or Methods section.

n/a	Confirmed	
☐	☒	The exact sample size (*n*) for each experimental group/condition, given as a discrete number and unit of measurement
☐	☒	A statement on whether measurements were taken from distinct samples or whether the same sample was measured repeatedly
☐	☒	The statistical test(s) used AND whether they are one- or two-sided<br>*Only common tests should be described solely by name; describe more complex techniques in the Methods section.*
☒	☐	A description of all covariates tested
☒	☐	A description of any assumptions or corrections, such as tests of normality and adjustment for multiple comparisons
☐	☒	A full description of the statistical parameters including central tendency (e.g. means) or other basic estimates (e.g. regression coefficient) AND variation (e.g. standard deviation) or associated estimates of uncertainty (e.g. confidence intervals)
☐	☒	For null hypothesis testing, the test statistic (e.g. *F*, *t*, *r*) with confidence intervals, effect sizes, degrees of freedom and *P* value noted<br>*Give P values as exact values whenever suitable.*
☒	☐	For Bayesian analysis, information on the choice of priors and Markov chain Monte Carlo settings
☒	☐	For hierarchical and complex designs, identification of the appropriate level for tests and full reporting of outcomes
☒	☐	Estimates of effect sizes (e.g. Cohen's *d*, Pearson's *r*), indicating how they were calculated

Our web collection on statistics for biologists contains articles on many of the points above.

Software and code

Policy information about availability of computer code

Data collection	Malvern iTC200 calorimeter (Malvern Instruments), ASTRA version 7.0.1 (Wyatt Technology Corporation), Topspin 2.1 and 4.0.9, Maestro version 11.0.014 (Schrödinger) with Desmond program (D. E. Shaw Research), ProteomeLab (Beckman Coulter), NIS-Elements Ar ver.5.21.00
Data analysis	Origin software version 7.0552, ASTRA version 7.0.1 (Wyatt Technology Corporation), Olivia software (v1,17.0), NMRPipe software (version 2012.090.12.09), SEDFIT version 15.01, Igor Pro version 8.04, Adaptive Poisson-Boltzmann Solver version 2.1, PyMol version 2.3.0.

For manuscripts utilizing custom algorithms or software that are central to the research but not yet described in published literature, software must be made available to editors and reviewers. We strongly encourage code deposition in a community repository (e.g. GitHub). See the Nature Research guidelines for submitting code & software for further information.

Data

Policy information about availability of data

All manuscripts must include a data availability statement. This statement should provide the following information, where applicable:

- Accession codes, unique identifiers, or web links for publicly available datasets
- A list of figures that have associated raw data
- A description of any restrictions on data availability

PDB: 5YVG, PDB: 4FDD
All data presented in the current study are available in the manuscript, supplement information or source data file.

Field-specific reporting

Please select the one below that is the best fit for your research. If you are not sure, read the appropriate sections before making your selection.

☒ Life sciences ☐ Behavioural & social sciences ☐ Ecological, evolutionary & environmental sciences

For a reference copy of the document with all sections, see nature.com/documents/nr-reporting-summary-flat.pdf

Life sciences study design

All studies must disclose on these points even when the disclosure is negative.

Sample size	We repeated each experiment three times and calculated the standard deviation. Our sample sizes are sufficient to ensure the credibility of results.
Data exclusions	No data was excluded.
Replication	Replicates are indicated in Figure Legends or Methods.
Randomization	Our study aimed to analyze molecular basis. Unlike other studies such as clinical trials, randomization was not relevant to our experimental design.
Blinding	Our study aimed to analyze molecular basis. Unlike other studies such as clinical trials, blinding was not relevant to our experimental design.

Reporting for specific materials, systems and methods

We require information from authors about some types of materials, experimental systems and methods used in many studies. Here, indicate whether each material, system or method listed is relevant to your study. If you are not sure if a list item applies to your research, read the appropriate section before selecting a response.

Materials & experimental systems

n/a	Involved in the study	
☐	☒	Antibodies
☐	☒	Eukaryotic cell lines
☒	☐	Palaeontology and archaeology
☒	☐	Animals and other organisms
☒	☐	Human research participants
☒	☐	Clinical data
☒	☐	Dual use research of concern

Methods

n/a	Involved in the study	
☒	☐	ChIP-seq
☒	☐	Flow cytometry
☒	☐	MRI-based neuroimaging

Antibodies

Antibodies used	Anti-Kapβ2 (Cat#sc-166127, 1:1000) was purchased from Santa Cruz. Anti-HA (Cat#561, 1:10000) was purchased from MBL. Goat Anti-Mouse IgG H&L (HRP) (Cat#ab6789, 1:10000) and Goat Anti-Rabbit IgG H&L (HRP) (Cat#ab6721, 1:10000) were purchased from abcam.
Validation	All antibodies were verified to be validated by the manufacturer for species specificity and the application used.<br>manufacturer's website of Anti-Kapβ2: https://datasheets.scbt.com/sc-166127.pdf<br>manufacturer's website of Anti-HA: https://ruo.mbl.co.jp/bio/dtl/dtlfiles/561_ver5.pdf<br>manufacturer's website of Goat Anti-Mouse IgG H&L (HRP): https://www.abcam.com/goat-mouse-igg-hl-hrp-ab6789.html<br>manufacturer's website of Goat Anti-Rabbit IgG H&L (HRP): https://www.abcam.com/goat-rabbit-igg-hl-hrp-ab6721.html

Eukaryotic cell lines

Policy information about cell lines

Cell line source(s)	HeLa cells were purchased from ATCC.
Authentication	Cell lines were not authenticated after they were received.
Mycoplasma contamination	Mycoplasma contamination testing was not performed after receipt of cell lines.
Commonly misidentified lines (See ICLAC register)	No commonly misidentified cell lines were used in this study.
