# Supplementary figures and images for: C9orf72-derived arginine-rich poly-dipeptides impede phase modifiers
Source: Nat Commun. 2021 Sep 6;12:5301. doi: 10.1038/s41467-021-25560-0 (PMC8421406; doi:10.1038/s41467-021-25560-0)

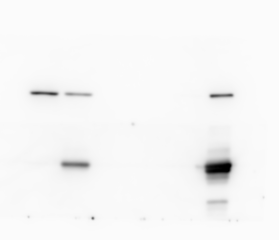

Supplement: Supplementary file 4 — source data [file 41467_2021_25560_MOESM4_ESM.zip › Source data file/Figure 2a original.tiff]

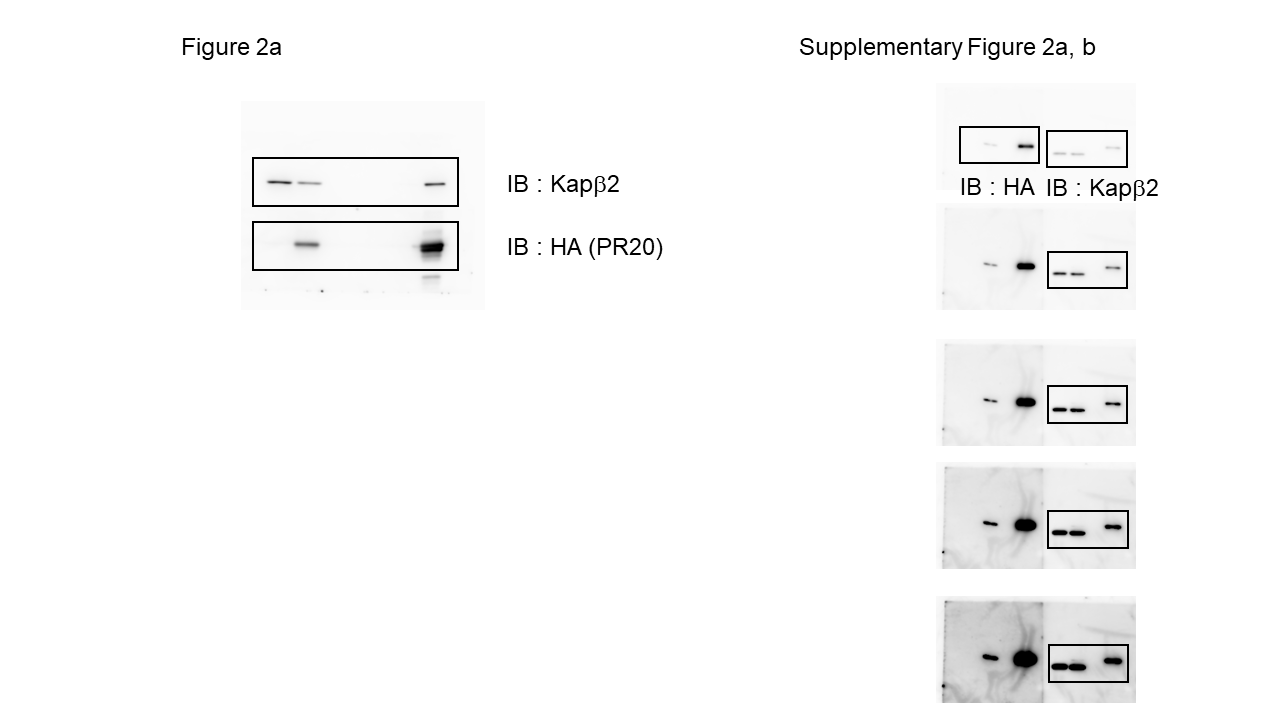

Supplement: Supplementary file 4 — source data [file 41467_2021_25560_MOESM4_ESM.zip › Source data file/Figure 2a, Supplementary Figure 2a.b original .tif]

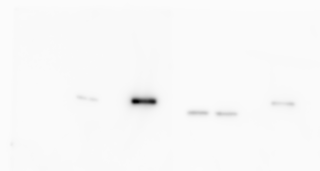

Supplement: Supplementary file 4 — source data [file 41467_2021_25560_MOESM4_ESM.zip › Source data file/Supplementary Figure 2a.b_(different exposure time)/Fig S2a, b original-1.tiff]

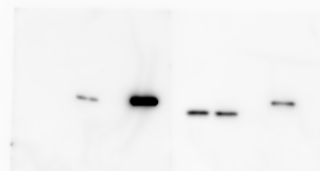

Supplement: Supplementary file 4 — source data [file 41467_2021_25560_MOESM4_ESM.zip › Source data file/Supplementary Figure 2a.b_(different exposure time)/Fig S2a, b original-2.tiff]

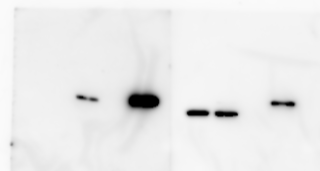

Supplement: Supplementary file 4 — source data [file 41467_2021_25560_MOESM4_ESM.zip › Source data file/Supplementary Figure 2a.b_(different exposure time)/Fig S2a, b original-3.tiff]

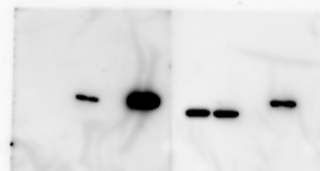

Supplement: Supplementary file 4 — source data [file 41467_2021_25560_MOESM4_ESM.zip › Source data file/Supplementary Figure 2a.b_(different exposure time)/Fig S2a, b original-4.tiff]
